# A Collision Tumor in the Breast Consisting of Invasive Ductal Carcinoma and Malignant Melanoma

**DOI:** 10.7759/cureus.23588

**Published:** 2022-03-28

**Authors:** John Grove, Miglena K Komforti, Laura Craig-Owens, Frank Beuerlein

**Affiliations:** 1 Pathology, Lincoln Memorial University DeBusk College of Osteopathic Medicine, Knoxville, USA; 2 Pathology, Cleveland Clinic Foundation, Cleveland, USA; 3 Pathology and Laboratory Medicine, Turkey Creek Medical Center, Knoxville, USA

**Keywords:** lymphovascular invasion, immunohistochemical stain, invasive ductal breast carcinoma, malignant melanoma metastasis, collision tumor

## Abstract

Collision tumors are rare neoplasms that consist of at least two different cell lineages at the same site. Given the many possible combinations that can occur, collision tumors, while rare, have been reported in multiple locations such as the stomach, bladder, and thyroid. Collision tumors are rarely found in breast tissue, with only a few cases reported in the literature. We herein report a unique case of a 79-year-old woman with a history of melanoma who presented with a left breast mass that was subsequently found to have invasive ductal carcinoma (IDC) and metastatic melanoma in the breast tissue. This is one of the first reported combinations of these two malignancies.

## Introduction

Breast cancer is the most common cancer in women and the second most common cancer in the world [1]. The prevalence of breast cancer in women ages 75-79 is 421 per 100,000 [2]. The prevalence of melanoma is lower than that of breast cancer, with roughly 25 per 100,000 in the United States [3]. Individually, these two cancers are diagnosed frequently; however, case reports of the two occurring in the same location as part of a collision tumor are rare. A collision tumor is the concrescence of two histologically unique neoplasms in one location [4,5]. What distinguishes a collision tumor from other complex tumors, such as a mixed tumor, is that a collision tumor has distinct borders between the two neoplasms. Only a few cases of collision tumors involving breast tissue have been reported, including cases of invasive ductal carcinoma (IDC) with squamous cell carcinoma, IDC with cutaneous melanoma, and IDC with MALT lymphoma [4,6,7].

## Case presentation

**Figure 1:**
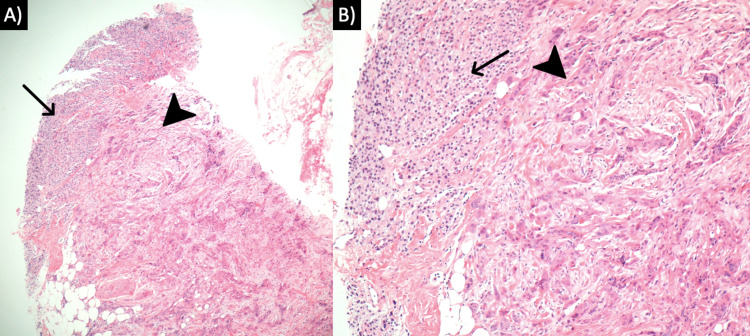
Hematoxylin and Eosin staining of collision tumor. (A) Low power view of breast tissue containing metastatic melanoma (arrow) adjacent to invasive ductal carcinoma (arrowhead). (B) High power view demonstrating the different morphologic presentation of the metastatic melanoma (arrow) and invasive ductal carcinoma (arrowhead).

**Figure 2:**
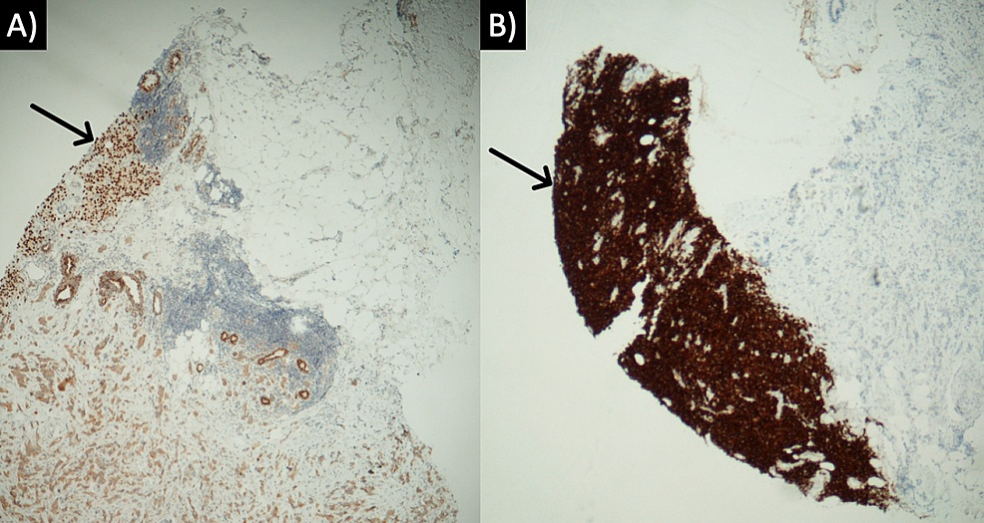
Immunohistochemical staining of collision tumor. (A) SOX10 stain of malignant melanoma and (B) pan-melanoma stain of metastatic melanoma.

**Figure 3:**
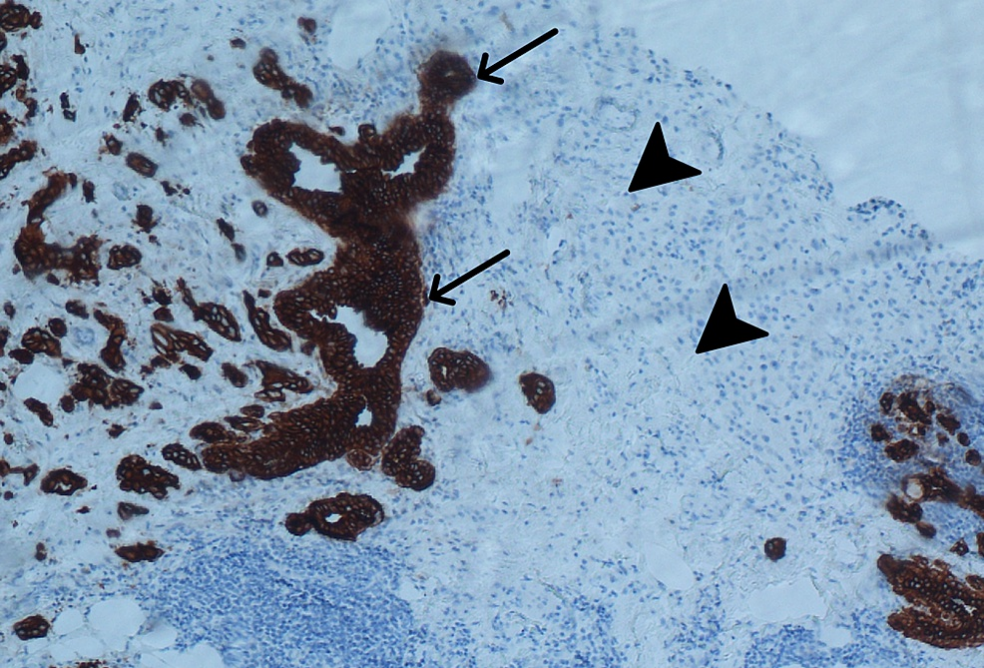
Positive AE1/AE3 immunohistochemical staining of the invasive ductal carcinoma component (arrows). The malignant melanoma is immediately adjacent (arrowheads) with no staining.

A 79-year-old female presented with a mass in the left breast following a recent diagnosis of metastatic melanoma identified in the right lobe of the liver. One week after the staging workup of the liver mass was completed, a breast lesion was identified on a physical exam. An ultrasound-guided core biopsy was performed. Microscopic examination revealed two histologically unique neoplasms immediately adjacent to one another (Figure 1). The first is IDC with focal squamous differentiation. Juxtaposed with this was a distinct malignant population of metastatic melanoma. The IDC was graded as provisional Nottingham grade III. The malignant melanoma component stained positive for SOX10 and Pan-melanoma markers (Figure 2) and was negative for AE1/AE3. The IDC tumor cells were positive for AE1/AE3 (Figure 3) and GATA3, but negative for estrogen receptor, progesterone receptor, and Her2 (1+). The patient was referred to a clinical oncologist for treatment.

## Discussion

Invasive ductal carcinoma is defined histologically as pleomorphic tumor cells organized into tubular formations and elastosis involving the walls of vessels and ducts [8]. Melanoma is the seventh most common form of cancer in women and the fifth most common in men [9]. Melanocytic malignancy can be distinguished from benign melanocytic lesions by the presence of cytological atypia, prominent nucleoli, and nuclear pseudoinclusions [10]. The immunohistochemical markers that help to establish a diagnosis of malignant melanocytic lesions include S100, HMB45, Melan A, and tyrosinase [10].

Collision tumors consisting of invasive ductal carcinoma and metastatic melanoma are rare and have only been reported a few times [7,11]. The most recent case report of a collision tumor consisting of melanoma and metastatic ductal carcinoma was in 2006, when the tumor was identified within a single axillary lymph node [11]. Breast cancer is the most common form of malignancy in women; however, it is very rare to find metastatic cancer in breast tissue [12]. The three most common distant metastases found in breast tissue are malignant melanoma, lymphoma, and lung cancer [12]. Understanding the morphology of these cancers is crucial in diagnosing collision tumors as clinical staging and therapeutic approaches might be altered for associated malignancies. This case promotes awareness among pathologists and oncologists as identifying both malignancies is essential in rare cases such as the one presented here. 

## Conclusions

A patient with a history of malignancies is alarming as potential metastases may occur. Here, we present a case of a rarely reported collision tumor - invasive ductal carcinoma and metastatic melanoma to the breast. The melanoma was first found in the right lobe of the liver and then subsequently found in a breast nodule. This case emphasizes the importance of recognizing collision tumors and the use of immunohistochemical staining in identifying tumor origins, thus preventing malignancies from being missed in the diagnosis. Further reporting of rare cases such as this one may reveal more instances of these occurrences in order to give an indication of their true incidence as few cases are reported in the literature.
